# One-phase phenol-free method for microRNA isolation from blood plasma

**DOI:** 10.1016/j.mex.2018.07.002

**Published:** 2018-07-06

**Authors:** A.N. Shirshova, D.A. Shamovskaya, U.A. Boyarskikh, N.E. Kushlinskii, M.L. Filipenko

**Affiliations:** a Institute of Chemical Biology and Fundamental Medicine SB RAS, Novosibirsk, Russia; b N. N. Blokhin National Medical Research Center of Oncology, Ministry of Health of the Russian Federation, Moscow, Russia

**Keywords:** One-phase phenol-free method for microRNA isolation from blood plasma, Short RNA, Isolation, Biofluids, One-phase phenol-free method for microRNA isolation from blood plasma

## Abstract

MicroRNA extraction is an essential procedure when discovering MicroRNA-based biomarkers and approaches. Here we describe a new method for microRNA isolation from human blood plasma, based on isopropanol precipitation from the one-phase lysate. We demonstrate that the use of more than four volumes of lysis buffer based on 5 M guanidine isothiocyanate prevents the formation of large, viscous, and hardly soluble precipitate. Applying widely used linear polyacrylamide (LPAA) as the only precipitating agent proved ineffective. At the same time, adding poly(A)RNA or tRNA with LPAA significantly increased the amount of microRNA obtained. Replacing β-mercaptoethanol with less volatile dithiothreitol in lysis buffer did not lead to a decrease in the yield. We compared the method proposed with miRNeasy Mini Kit (Qiagen) for isolation of microRNA from human blood plasma. MicroRNA yield was evaluated by the difference in median Ct values obtained for exogenous cel-238 and endogenous microRNA-21 cDNA amplification. For both tested microRNA, the precipitation from one-phase lysate provided better recovery with lower Ct values (Δ median Ct 4.94 for cel-238, р = 1,0E-04 and Δ median Ct 2.18 for microRNA-21, р = 9,0E-04). Thus, the method we described showed high yield and operating convenience because it can be applied without special equipment.

## Graphical abstract

**Figure:**
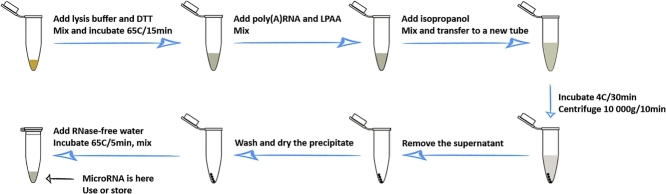

## Specifications Table

**Table:** 

Subject area	Biochemistry, Genetics and Molecular Biology
More specific subject area	MicroRNA isolation
Protocol name	One-phase phenol-free method for microRNA isolation from blood plasma
Reagents/tools	- lysis buffer (5 M guanidine isothiocyanate, 0.75 M NaCl, 0.5 % SDS, 5 % Triton X100, 20 mM TrisHCl, pH = 8) - 1 M DTT - 1 μg/μl poly(A)RNA (or 1 μg/μl yeast tRNA) - 5 μg/μl LPAA - isopropanol - 1 wash buffer (60 % isopropanol, 10 mM TrisHCl, pH = 8.0) - 2 wash buffer (75 % ethanol, 10 mM TrisHC,l pH = 8.0) - RNase-free water
Experimental design	To select the best reagents composition 6 comparative isolations from 8 plasma samples were performed. The following variables were tested: the ratio of the plasma sample volume to the lysis buffer volume, the molarity of NaCl in the lysis buffer, the effect of replacement of β-mercaptoethanol by dithiothreitol and effect of the addition of LPAA and RNA (poly(A)RNA and tRNA) as coprecipitator. As a result, we got the protocol that showed better recovery efficiency: 4 volumes of lysis buffer containing 0.75 M NaCl and adding 2.5 μM DTT, 10 μg poly(A)RNA, 50 μg of LPAA per sample. In comparison with miRNeasy Mini Kit, our protocol showed better yield (Δ median Ct 4.94 for cel-238, р = 1,0E-04 and Δ median Ct 2.18 for microRNA-21, р = 9,0E-04).
Trial registration	–
Ethics	All donors were familiar with the content of the work and signed informed consent.

## Value of the Protocol

• The method yields high amounts of high-quality miRNA that is ready for use in any downstream application, including qRT–PCR.
• The method does not use phenol, chloroform, β-mercaptoethanol and other toxic reagents, that should be carefully handled.
• The cost of sample isolation is low.

## Description of protocol

MicroRNAs are new potential markers of a wide variety of biological conditions [1,2]. It is a challenge to discover a new markers, and isolation methods have significant role. The method for microRNA isolation from plasma by isopropanol precipitation from one-phase lysate without the use of phenol was proposed and tested. To select the best reagents composition 6 comparative isolations from 8 plasma samples were performed. In comparison with miRNeasy Mini Kit, method proposed showed better yield (Δ median Ct 4.94 for cel-238, р = 1,0E-04 and Δ median Ct 2.18 for microRNA-21, р = 9,0E-04).

## Method details

### Preparation of plasma

Plasma samples of 8 healthy donors were used. All donors were familiar with the content of the work and signed informed consent. Approximately 10 ml of venous blood was collected into the tube containing EDTA, thoroughly but softly mixed and centrifuged for 10 min at 1600 g at room temperature. The resulting supernatant (approximately 4–5 ml) was carefully selected without capturing the precipitate and transferred into the 15 ml tubes. The supernatant was centrifuged for the second time for 10 min at 1600 g at room temperature and divided into 50 μl aliquots and transferred into 2 ml tubes, frozen and stored at -80 °C for future use.

### MicroRNA extraction protocol

1 Heat the lysis buffer (5 M guanidine isothiocyanate, 0.75 M NaCl, 0.5% SDS, 5% Triton X100, 20 mM TrisHCl, pH = 8) to 65 °C. Add 200 μl of hot lysis buffer, and 2,5 μl of 1 M DTT per 50 μl of plasma;
2 Mix thoroughly and incubate for 15 min at 65 °C;
3 Add 10 μl of poly(A)RNA (1 μg/μl) and 10 μl of LPAA (5 μg/μl). Add a spiked-in synthetic RNA, if it is required;
4 Mix thoroughly;
5 Add 400 μl of isopropanol;
6 Mix, drop the droplets by short centrifugation, and transfer the whole sample volume into a new 2 ml tube without touching the walls. Do not rotate tubes;
7 Incubated for 30 min at +4 °C;
8 Centrifuge for 10 min at 10,000 g;
9 Remove the supernatant carefully;
10 Wash the precipitate with 400 μl of wash buffer 1 (60% isopropanol, 10 mM TrisHCl pH = 8.0);
11 Wash the precipitate with 400 μl of wash buffer 2 (75% ethanol, 10 mM TrisHCl pH = 8.0);
12 Remove wash buffer fully and dry precipitate for 10 min at 37 °C;
13 Add 50 μl of RNase-free water and incubated for 5 min at 65 °C on the thermoshaker;
14 Use or store the microRNA at −80 °C.

## Method validation

To select the best reagents composition 6 comparative isolates from plasma samples of 8 healthy donors were performed using method described above, and following variables of the method were tested:

- ratio of the plasma sample volume to the lysis buffer volume;
- molarity of NaCl in the lysis buffer: 0,5 M or 075 M NaCl;
- replacement of β-mercaptoethanol by dithiothreitol: 143 μM β-mercaptoethanol or 2,5 μM DTT per sample;
- addition following coprecipitators: 50 μg LPAA, or 50 μg LPAA + 10 μg of tRNA, or 50 μg LPAA + 10 μg poly(A)RNA, or 10 μg poly(A)RNA per sample per sample.

Each plasma sample was analyzed individually, not as a pooled sample.

As a comparison, miRNeasy Mini Kit was used (Qiagen, cat No./ID: 217004). The isolation was carried out according to the manufacturer's protocol.

### MicroRNA-specific reverse transcription (RT)

Complementary DNA was obtained using specific RT stem-loop primer (Table 1). The 3′ end tail of RT-primer has 6 specific nucleotides which complementing 3′ end of mature microRNA sequence followed by sequences complementing reverse PCR primer and the dye-labeled probe. Stem structure might enhance the thermal stability of the microRNA–RT primer heteroduplex. RT reaction contained 2.7 μl of extracted RNA, 0.8 μl (10 μM) stem-loop RT primer, 4 μl (2X) RT buffer, 200 U MMLV in a volume of 8 μL. Reverse transcription was carried out at 16 °C for 30 min and 42 °C for 30 min, and 95 °C for 5 min for the inactivation of the reaction. Resulting mixtures were diluted 7 times with RNase-free water to avoid PCR inhibition.

**Table 1:** Sequences of primers and probes used in RT and PCR.

	Sequence
microRNA-21	
RT stem-loop primer	5’-GTCGTATCCAGTGCAGGGTCCGAGGTATTCGCACTGGATACGACTCAACA-3’
PCR primers and due-labeled probe	U 5’-GCCCGCTAGCTTATCAGACTGAT-3’ R 5’-GTGCAGGGTCCGAGGT-3’ 5’-HEX-GCACTGGATACGACTCAACA-BHQ2-3’
cel-238	
RT stem-loop primer	5’-GTCGTATCCAGTGCAGGGTCCGAGGTATTCGCACTGGATACGACTCTGAA-3’
PCR primers and due-labeled probe	U 5’-TTTGTACTCCGATGCC-3’ R 5’- GTGCAGGGTCCGAGGT-3’ 5’-ROX-TTCGCACTGGATACGACTCTGAA-BHQ2-3’

### Quantification of microRNA by real-time PCR (PCR)

cDNA was amplified using specific forward primer, dye-labeled probe, and universal reverse primer. CFX96 Real-Time PCR System (Bio-Rad) was used. PCR reaction contained 2 μl cDNA, 300 nM PCR primers and 100 nM dye-labeled probe (Table 2), 8 μl (2X) PCR buffer and 0.5 U Taq-polymerase with hot-start in a volume of 16 μl. The reactions were incubated in a 96-well plate at 95 °C for 15 min, followed by 40 cycles of 95 °C for 10 s and 57 °C for 30 s. All reactions were run in duplicate.

**Table 2:** Quantification cycle values for microRNAs isolated by 6 methods that were compared. Statistical data are given for each comparisons of reagent compositions.

		median Ct	lower quartile-upper quartile	median Ct	lower quartile-upper quartile	Δ median Ct	p-value
1		0,5 M NaCl (+ LPAA + β-mercaptoethanol)		0,75 M NaCl (+ LPAA + β-mercaptoethanol)			
	cel-238	20.45	20.35–20.69	20.04	19.97–20.22	0.41	0.001
	microRNA-21	26.46	26.65–26.71	26.02	25.97–26.22	0.44	0.018
2		β-mercaptoethanol (+ 0,5 M NaCl + LPAA)		dithiothreitol (+ 0,5 M NaCl + LPAA)			
	cel-238	20.45	20.35–20.69	20.43	20.04–20.69	0.02	0.334
	microRNA-21	26.46	26.26–26.71	26.49	26.31–26.61	0.03	0.713
3		without LPAA (+ 0,75 M NaCl + DTT)		LPAA (+ 0,75 M NaCl + DTT)			
	cel-238	20.61	20.61–21.3	20.47	20.26–21.09	0.14	0.316
	microRNA-21	30.84	30.71–30.99	29.85	29.61–30.02	0.99	0.014
4		LPAA (+ 0,75 M NaCl + DTT)		tRNA + LPAA (+ 0,75 M NaCl + DTT)			
	cel-238	20.31	20.13–20.61	18.81	18.67–19.17	1.5	4.8E-06
	microRNA-21	30.19	29.77–30.37	27.63	27.39–27.83	2.56	0.018
5		LPAA (+ 0,75 M NaCl + DTT)		poly(A)RNA + LPAA (+ 0,75 M NaCl + DTT)			
	cel-238	20.31	20.13–20.61	17.18	17.07–17.41	3.13	3.0E-06
	microRNA-21	30.19	29.77–30.37	27.16	26.94–27.63	3.03	0.018
6		poly(A)RNA (+ 0,75 M NaCl + DTT)		poly(A)RNA + LPAA (+ 0,75 M NaCl + DTT)			
	cel-238	17.73	17.20–18.12	17.18	17.07–17.41	0.55	0.005
	microRNA-21	27.96	27.57–28.30	27.16	26.94–27.63	0.8	0.018
7		method proposed (+ 0,75 M NaCl + DTT + poly(A)RNA + LPAA)		miRNeasy Mini Kit			
	cel-238	17.18	17.07–17.41	22.12	22.04–22.44	4.94	1.0E-04
	microRNA-21	27.16	26.94–27.63	29.34	28.95–29.84	2.18	9.0E-04

### Assessment of microRNA purity

The microRNA purity was assessed using the relative absorbance ratio at A260/280 (spectrophotometer NanoDrop Technologies, Wilmington, DE, USA) and by RT-PCR of RNA serial dilutions.

### Data analysis

The data were expressed as the median threshold cycle (median Ct), the lower quartile and upper quartiles and the difference in the median Ct (Δ median Ct) of microRNA-21 and cel-238. The Ct was defined as the cycle number at which the fluorescence passes the fixed threshold. Statistical comparison of the data was performed by Mann-Whitney *U* test P-values less than 0.05 were considered statistically significant. All of the statistical tests were carried out using SPSS 12.0 (SPSS Inc., Chicago, IL, USA).

## Results

The method for microRNA isolating from plasma by isopropanol precipitation from a one-phase lysate without the use of phenol was proposed and tested. The following variables were tested: the ratio of the plasma sample volume to the lysis buffer volume and the molarity of NaCl in the lysis buffer. Also, the effect of replacement of β-mercaptoethanol by dithiothreitol and effect of the addition of LPAA and RNA (tRNA and poly(A)RNA) as coprecipitator were checked.

### The ratio of the volume of the plasma sample to the lysis buffer

Commonly, 2-2,5 volumes of lysis buffer containing 4–6 M guanidine isothiocyanate per 1 vol of plasma are used. We have increased the volume of the lysis buffer to 4 volumes, assuming that this would eliminate the precipitation of lipids and other obstructive plasma components. In some cases, we observed large viscous precipitates when less than 4 volumes were used. After increasing the lysis buffer volume, such type of precipitate was not observed.

### The molarity of NaCl

To investigate whether the molarity of NaCl affects microRNA yield, microRNA was isolated from plasma samples of 8 healthy donors using 0.75 M and 0.5 M NaCl. MicroRNA was reverse transcribed an amplified as described above. The data shown in Table 2 demonstrate that 0.75 M NaCl slightly increased the yield of microRNA. Similar Δ median Ct volumes of both cel-238 and microRNA-21 (Δ median Ct 0.4) were obtained.

### Replacement of β-mercaptoethanol with dithiothreitol

Since one of the priorities of the development of the method was the refusal to use toxic reagents, the widely used β-mercaptoethanol was replaced by dithiothreitol, which is practically odorless, resulting in no significant difference. Similar result is described earlier [3].

### Addition of LPAA as a coprecipitator

LPAA is widely used as coprecipitator for isolation of nucleic acids [4]. In our experiments, slightly better result was obtained with the addition 50 μg of LPAA than without for microRNA-21, and there was no difference for cel-238 (Δ median Ct 0.14 of cel-238 and 0.99 of microRNA-21).

### Addition of LPAA in combination with tRNA or poly(A)RNA as the coprecipitators

In addition to LPAA, 5–10 μg of total MS2 bacteriophage or yeast RNA or tRNA or poly(A)RNA per sample is widely used as a coprecipitator to increase the RNA yield [5]. To investigate the recovery of microRNA with and without using RNA as coprecipitator, microRNA was isolated from 8 plasma samples, and 50 μg of LPAA and 10 μg of tRNA or poly(A)RNA was added to test isolation. Significantly better results were obtained, when adding RNA. In the case of tRNA, Δ median Ct was 1.5 of cel-238 and 2.56 of microRNA-21. In the case of poly(A)RNA, the difference was greater: Δ median Ct was 3.13 of cel-238 and 3.03 of microRNA-21. We assume that higher tRNA concentration can provide better results.

### Addition poly(A)RNA or poly(A)RNA in combination with LPAA as the coprecipitators

Since we did not get much better results when LPAA was added relative to samples without carrier, we compared the isolation efficiency when poly(A)RNA was added with and without LPAA. We obtained the same results – addition of LPAA provided slightly better results (Δ median Ct 0.55 of cel-238 and 0.8 of microRNA-21). In addition, in the presence of LPAA precipitations are seen a little better and better attached to the bottom of the tube during processing.

### Comparison with miRNeasy Mini Kit

To compare the method proposed we used miRNeasy Mini Kit (Qiagen, cat No./ID: 217004). Our protocol was compared in modification that showed greater efficiency: 4 volumes of lysis buffer containing 0.75 M NaCl and 2.5 μM DTT, 10 μg poly(A)RNA, 50 μg of LPAA. Using our method and miRNeasy Mini Kit, microRNA was isolated simultaneously from 8 plasma samples, reverted and amplified as described above. The miRNeasy Mini Kit protocol offers to elute the microRNAs from silica-spin columns in the volume of 14 μl. We increased the volume to 50 μl because in the method proposed we used 50 μl. Thus, the conditions of isolation were the same for both methods. We obtained the greater yield of microRNA using the protocol proposed for both microRNA (Δ median Ct 4.94 of cel-238 and 2.18 of microRNA-21, the differences were statistically significant).

Differences in Ct values of microRNA-21 from Table 2 obtained for the first two comparisons (molarity of NaCl and dithiothreitol tests) and further test due to usage of different plasma samples.

We did not evaluate RNA yield by spectroscopy or fluorometry methods due to usage of poly(A)RNA or tRNA carriers that change the real content of microRNA.

### The purity of microRNA

The RNA purity was assessed using the relative absorbance ratio at A260/280. As was expected due to high content of adenine within the poly(A)RNA in samples obtained with the method proposed we got high A260/280 ratio: 2.35–2.6. On the other hand, that indicated that isolated RNAs were relatively free of proteins. For RNA samples obtained with Qiagen kit, A260/280 ratio ranged from 1.8–1.95. Since in the method proposed some impurities can precipitate with RNA simultaneously, we further tested whether those impurities obstruct the RT and PCR reactions. For 2 pooled RNA samples (8 samples obtained with the method proposed and 8 obtained with Qiagen kit) serial dilutions of 5 points were prepared and RT-PCR was carried out. We got the same PCR efficiency (E) and sufficient correlation coefficient (R^2^) for both pooled samples for both microRNA: cel-238 (method proposed E = 102.0, R^2^ = 0.995; Qiagen kit E = 102.8, R^2^ = 0.998) and microRNA-21 (method proposed E = 99.3, R^2^ = 0.997; Qiagen kit E = 100.2, R^2^ = 0.999). Thus, impurities in the microRNA samples obtained with our method did not obstruct the RT and PCR reactions.

## Discussion

We proposed and evaluated method for microRNA isolation from human blood plasma based on isopropanol precipitation from the one-phase lysate. To select the best reagents composition 6 comparative isolations we performed. As a result, we got the protocol that showed better recovery efficiency: 4 volumes of lysis buffer containing 0.75 M NaCl and adding 2.5 μM DTT, 10 μg poly(A)RNA, 50 μg of LPAA per sample. In comparison with miRNeasy Mini Kit, this protocol showed better yield (Δ median Ct 4.94 for cel-238, р = 1,0E-04 and Δ median Ct 2.18 for microRNA-21, р = 9,0E-04). Potential disadvantage of our method could be a sufficiently large volume of water (50 μl) needed to dissolve the precipitate for some plasma samples. miRNeasy Mini Kit could be more effective in preparation of more concentrated miRNA samples due to possibility to eluate microRNA from column using low volume (as low as 14 μl according to manual). On the other hand, there is some probability that small elution volume is not enough to elute the entire microRNA from the column [6] and leads to loss of yield.

It is known that the use of RNA as a coprecipitator improves the microRNA isolation efficiency from plasma samples [5]. In our experiments, we also got the better result when tRNA or poly(A)RNA was added. Drawback of this approach could be some restriction for usage of microRNA detection method that include polyadenylation step or adaptor ligation step due to presence of large amount of non-specific RNA in sample. The use of tRNA or poly(A)RNA as coprecipitator further improved the yield and made the process more stable and reproducible.

Unfortunately, guanidine isothiocyanate crystallizes on the tube cap, and therefore it is necessary to transfer the sample to the new tube. We hope to optimize this and avoid crystallization, and this will significantly shorten the time of samples preparation.

This study has several limitations. Undoubtedly, the main limitation of this work is the small namber of the samples studied and the lack of validation of the protocol proposed on plasma samples with different quality and obtained by different methods. As well as we measured only single endogenous and exogenous microRNA in plasma samples. Moreover other human biofluids could be important potential sources of biomarkers and are the subject of our future study using approach proposed.
